# Iatrogenic Type A Aortic Dissection: Challenges and Frontiers—Contemporary Single Center Data and Clinical Perspective

**DOI:** 10.1055/s-0042-1756670

**Published:** 2022-12-15

**Authors:** Konstantin von Aspern, Sergey Leontyev, Christian D. Etz, Josephina Haunschild, Martin Misfeld, Michael A. Borger

**Affiliations:** 1 University Department for Cardiac Surgery, Leipzig Heart Center, Leipzig, Saxony, Germany; 2 Department of Cardiothoracic Surgery, Royal Prince Alfred Hospital, Sydney, New South Wales, Australia; 3 Department of Cardiac Surgery, Sydney Medical School, University of Sydney, Sydney, New South Wales, Australia; 4 Institute of Academic Surgery, Royal Prince Alfred Hospital, Sydney, New South Wales, Australia; 5 The Baird Institute of Applied Heart and Lung Surgical Research, Sydney, New South Wales, Australia

**Keywords:** iatrogenic aortic dissection, Type A, complications associated with cardiac surgery

## Abstract

Iatrogenic aortic dissection (IAD) is a rare but devastating complication in cardiac surgery and related procedures. Due to its rarity, published data on emergency surgery following IAD are limited. Herein, we discuss IAD occurring intra- and postoperatively, including those occurring during transcatheter aortic valve replacement and cardiac catheterization, and present benchmark data from our consecutive, single-center experience. We demonstrate changes in patient characteristics, surgical approaches, and outcomes over a 23-year period.

## Introduction

Iatrogenic aortic dissection (IAD) is a rare but devastating complication associated with cardiac surgery and related procedures. 1 2 3 Due to the rarity of this condition, published data on emergency surgery following IAD are limited to small case series, registry data, and meta-analyses. 2 4 5 6 IAD as a complication of cardiac surgery has already been reported as early as 1960. 7 During these early years of cardiopulmonary bypass (CPB)-assisted operations, IAD was a more common and well-known complication with an incidence of approximately 0.8% and was associated with a high mortality rate of 45 to 50%. 1 2 8 Retrograde arterial cannulation via the femoral vessels was a common method for establishing CPB in the earlier reported cases. Ascending aortic cannulation as the preferred technique for CPB contributed to a decrease in observed IAD. 2 However, with an increasing focus on minimally invasive procedures in modern cardiac surgery (e.g., lateral minithoracotomies for aortic and mitral valve surgery), retrograde perfusion techniques are being again utilized more frequently.

According to recent reports, the incidence of IAD now ranges from 0.06 to 0.23%. 1 4 5 9 The proportion of all surgically treated Type A dissection patients that suffered from IAD is estimated to be approximately 5%. 6 10 With regard to cardiac catheterization, IAD is less frequent with a reported incidence between 0.01 and 0.02%. 1 11

Regardless of the advances in cardiac surgery over the last decades—which have led to a substantial decrease in overall surgery-related mortality—perioperative mortality for IAD remains high. Reports from current case series and registry data estimate the perioperative mortality rate of IAD to be 30 to 50%, which is substantially higher than surgery for spontaneous Type A dissection. 1 2

It has been shown that patients suffering from IAD during the early postoperative course are subject to higher mortality compared with patients sustaining dissections intraoperatively. 1 2 Recent reports therefore direct particular focus on the timely diagnosis of this devastating complication, involving all members of the surgical team to minimize its incidence and improve outcome. With regard to the incidence of postoperative IAD, some authors hypothesize that the number of unknown/undetected cases may be significantly higher owing to the low number of routinely performed autopsies following sudden deaths after cardiac surgery. 2 12 13

Herein, we focus on IAD occurring during (1) various cardiac surgery procedures (including transcatheter aortic valve replacement, TAVR), (2) the early postoperative period, and (3) other related procedures such as cardiac catheterization. By analyzing our institutional database—representing the largest consecutive single-center cohort to date—we also present current benchmark data on this important complication. Changes in patient characteristics, surgical approaches, and early outcomes after IAD over a 23-year period are investigated by comparing current data with IAD patients from earlier reports.

## Materials and Methods

### Patient Population and Definitions

Of all patients operated on at our center between March 1995 and January 2019 ( *n* = 87,524), we identified 92 patients (0.1%) who underwent emergency surgery for IAD. These patients were included and retrospectively analyzed. Immediate surgical aortic repair was performed in all cases. The cause of IAD was documented and classified as follows: (1) intraoperative, (2) postoperative, and (3) related to cardiac catheterization. The location and extent of the dissection was confirmed during surgery with transesophageal echocardiography (TEE) and direct inspection. In case of suspected postoperative IAD in stable patients, computed tomography (CT) was performed prior to reoperation. In patients with catheter-induced IAD, the diagnosis was confirmed by aortic angiography at the time of catheterization.

Intra- and postoperative IAD was defined as all confirmed dissection cases during or within 1 hour after surgery and thereafter, respectively. Early mortality was defined as all-cause mortality at 30 days or during index hospitalization. Preoperative patient variables were defined as previously published. 1 Coronary malperfusion consequential to IAD was clinically assumed by the discretion of the respective medical team taking into consideration the following diagnostic variables: (1) typical changes on electrocardiogram, (2) new onset low cardiac output with wall motion dysfunction, and (3) impact on coronary perfusion diagnosed by angiogram.

To investigate changes in patient's characteristics as well as perioperative management and outcome over time, the cohort was divided into two groups for comparison as follows: (1) patients operated on during the early (1995–2010, *n* = 48) and (2) the more recent (after 2010, *n* = 44) time periods.

### Surgical Management

According to our institutional approach for all patients with Type A dissection, aortic repair included resection of the intimal tear (when located in the ascending aorta or aortic arch) and as much of the dissected aorta as possible, without excessively increasing circulatory arrest times. Details of our surgical approach have been previously described in detail. 1 14 Changes in surgical management over the study period (1995–2019) including arterial cannulation, cerebral perfusion, temperature management, and extent of distal aortic repair are discussed in more detail below (see Results section “Changes over Time”).

### Statistical Analysis

Data were imported to Excel (Microsoft Excel for Mac 2011; Redmond, Washington, WA) and Statistical Package for the Social Sciences (SPSS Statistics 22.0; Chicago, IL) for description and analysis. Continuous variables are expressed as mean ± standard deviation and categorical data as proportions. Categorical data were compared using the Chi-square test or Fisher's exact test where appropriate. Continuous variables were assessed for normal distribution using the Shapiro–Wilks test, and data were compared using Student's *t* -test or Wilcoxon–Mann–Whitney test, where appropriate. A *p* -value < 0.05 was considered statistically significant. Variables were tested for associations with adverse outcomes using a univariable regression model. Risk factors for early outcomes are expressed as odds ratios (ORs) with a 95% confidence interval (CI). Multivariable logistic regression analysis was performed to determine independent predictors of mortality, using those variables with a statistical association ( *p* < 0.20) or known/ suspected pathophysiological effect on early survival in the model. Risk factor analyses were pooled over the entire surveillance period due to the limited number of patients and events. All tests were performed as two-sided at a significance level of 5%.

## Results

### Patient Characteristics

The mean age of all patients was 69 ± 12 years and 57% ( *n* = 52) were female. Overall, patients had a typical distribution regarding cardiovascular risk factors. The mean left ventricular ejection fraction (LVEF) was 52 ± 13% with 9% ( *n* = 8) of patients presenting with the LVEF of less than 30%. All clinical characteristics are presented in Table 1 , along with a comparison of patient characteristics in the two time periods.

**Table 1:** Preoperative patient characteristics

	Entire cohort Mean ± SD/ *n* (%)	1995–2010 Mean ± SD/ *n* (%)	2010–2019 Mean ± SD/ *n* (%)	*p* -Value
Number of patients	92	48	44	
Age (y)	68.6 ± 12	65.4 ± 13	72.0 ± 9	**0.007**
Female	52 (57)	24 (50)	28 (64)	0.212
LVEF	51.5 ± 13	50.7 ± 12	52.4 ± 14	0.541
LVEF < 30%	8 (9)	5 (10)	3 (7)	0.716
NYHA III/IV	50 (54)	23 (48)	27 (61)	0.215
COPD	7 (8)	2 (4)	5 (11)	0.253
PVD	16 (17)	11 (23)	5 (11)	0.176
pHT	10 (11)	4 (8)	6 (14)	0.511
CRF	43 (47)	24 (50)	19 (43)	0.537
Preoperative dialysis	2 (2)	1 (2)	1 (2)	1.000
Neurological dysfunction	9 (10)	5 (10)	4 (9)	1.000
Diabetes mellitus	24 (26)	15 (31)	9 (20)	0.342
Previous cardiac surgery	23 (25)	14 (29)	9 (20)	0.470
Aortic aneurysm	19 (21)	10 (21)	9 (20)	1.000
Coronary malperfusion	30 (33)	13 (27)	17 (39)	0.271

Abbreviations: COPD, chronic obstructive pulmonary disease; CRF, chronic renal failure; LVEF, left ventricular ejection fraction; NYHA, New York Heart Association functional class; pHT, pulmonary hypertension; PVD, peripheral vascular disease; SD, standard deviation.

### Incidence and Cause of Iatrogenic Dissection

A total of 64 patients (69.6%) suffered IAD during or shortly after cardiac surgery (including TAVR), while 28 (30.4%) presented with IAD during cardiac catheterization. The total incidence of IAD for cardiac surgical procedures was 0.07%. In patients who underwent TAVR, the incidence was comparable at 0.10% ( *n* = 6, *p* = 0.311). The type of primary surgical procedure (index surgery) is listed in Table 2 . IAD occurred intraoperatively in 53 cardiac surgery patients and postoperatively in 11. In case of postoperative IAD, the median time from index operation to IAD diagnosis was 2 days (range: 2–120 days).

**Table 2:** Iatrogenic aortic dissection details

	Entire cohort * n* (%)	1995–2010 *n* (%)	2010–2019 *n* (%)	*p* -Value
*Index surgery* :	64 (70)	35 (55)	29 (45)	0.377
CABG	17 (27)	11 (31)	6 (21)	0.402
Aortic valve (AV)	10 (16)	5 (14)	5 (17)	1.000
Mitral valve (MV)	16 (25)	9 (26)	7 (24)	1.000
MV (MIS)	12 (19)	6 (17)	6 (21)	0.757
Aortic surgery	3 (5)	3 (9)	0 (0)	0.245
Combination	12 (19)	6 (17)	6 (21)	0.757
TAVR	6 (9)	1 (3)	5 (17)	0.100
*Intraoperative IAD* :	53 (58)	31 (63)	22 (39)	0.206
Arterial cannulation	22 (42)	12 (39)	10 (45)	0.812
Cardioplegia cannulation	13 (25)	9 (29)	4 (18)	0.237
Aortic clamp site	6 (11)	4 (13)	2 (9)	0.679
Other locations	6 (11)	4 (13)	2 (9)	0.679
Unknown	6 (11)	5 (16)	1 (5)	0.206
*Postoperative IAD* :	11 (12)	5 (10)	6 (14)	0.752
Arterial cannulation	2 (18)	2 (40)	0 (0)	0.496
Cardioplegia cannulation	4 (36)	0 (0)	4 (67)	**0.049**
Aortic anastomosis	1 (9)	1 (20)	0 (0)	1.000
Unknown	4 (36)	2 (40)	2 (33)	1.000
*Cardiac catheterization* :	28 (30)	13 (27)	15 (34)	0.503
Right coronary ostium	14 (50)	9 (69)	5 (33)	0.128
Left coronary ostium	3 (11)	0 (0)	3 (20)	0.226
Aortic root	4 (14)	2 (15)	2 (13)	1.000
Ascending aorta	3 (11)	1 (8)	2 (13)	0.605
Aortic arch	2 (7)	1 (8)	1 (7)	1.000
Unknown	2 (7)	0 (0)	2 (13)	0.226
*TAVR IAD* :	6 (7)	1 (2)	5 (11)	0.100
* TF-TAVR* :	3 (50)	0 (0)	3 (60)	1.000
Aortic root	2 (33)	0 (0)	2 (40)	1.000
Ascending aorta	1 (17)	0 (0)	1 (20)	1.000
* TA-TAVR* :	3 (50)	1 (100)	2 (40)	1.000
Aortic root	1 (17)	1 (100)	0 (0)	0.167
Ascending aorta	2 (33)	0 (0)	2 (40)	1.000

Abbreviations: CABG, coronary artery bypass graft; IAD, iatrogenic aortic dissection; MIS, minimally invasive surgery; TA, transapical; TAVR, transcatheter aortic valve replacement; TF, transfemoral.

In surgical patients, IAD was most frequently associated with arterial ( *n* = 24, 37.5%) and cardioplegia ( *n* = 17, 26.6%) cannulation. In case of IAD due to arterial cannulation, the majority of patients were cannulated centrally during the index operation ( *n* = 20, 83.3%). Four patients were operated on using peripheral (femoral) cannulation with subsequent retrograde dissection propagation ( *n* = 4, 16.7%).

In TAVR patients, IAD was observed equally often in the ascending aorta ( *n* = 3) and aortic root ( *n* = 3). In patients who suffered from IAD postoperatively, the most frequent location was the cardioplegia cannulation site after minimally invasive mitral valve repair ( *n* = 4).

During cardiac catheterization, IAD occurred most commonly at the right coronary ostium ( *n* = 14, 50.0%).

Specific information on IAD characteristics is also presented in Table 2 .

### Extent of Surgical Repair and Operative Details

Most patients underwent replacement of the ascending aorta and partial or total arch ( *n* = 52, 56.5%). An isolated ascending aortic replacement was performed in 21 patients (22.8%), all of which had the dissection limited to the ascending aorta and/or root. Root replacement was necessary in 19 patients, (20.7%), and in 30 patients (32.6%) aortic valve replacement was performed. Fifty percent of patients ( *n* = 46) required concomitant coronary bypass surgery.

The mean myocardial ischemic time was 102 ± 43 minutes, and CPB time was 205 ± 80 minutes. In case of hemiarch replacement with an open distal anastomosis, the circulatory arrest time was 17 ± 10 minutes. For a total arch procedure, the arrest time was 24 ± 12 minutes. All intraoperative details are presented in Table 3 .

**Table 3:** Extent of aortic repair and other intraoperative data

	Entire cohort Mean ± SD/ *n* (%)	1995–2010 Mean ± SD/ *n* (%)	2010–2019 Mean ± SD/ *n* (%)	*p* -Value
*Operative extent* :				
Aortic root/ascending aorta replacement	19 (21)	11 (23)	8 (18)	0.615
Aortic arch	8 (42)	4 (36)	4 (50)	0.658
Isolated ascending aorta	21 (23)	14 (29)	7 (16)	0.145
Ascending aorta + arch	52 (57)	23 (48)	29 (66)	0.096
* All aortic arch procedures* :	60 (65)	27 (56)	33 (75)	0.080
Hemiarch replacement	16 (17)	7 (15)	9 (20)	0.724
Total arch replacement	44 (48)	20 (42)	24 (55)	0.523
*Concomitant procedures* :				
AV replacement	30 (33)	10 (21)	20 (45)	**0.015**
MV repair or replacement	17 (18)	12 (25)	5 (11)	0.112
CABG ( *n* —%)	46 (50)	24 (50)	22 (50)	1.000
CABG + MV surgery	10 (11)	5 (10)	5 (11)	1.000
*Intraoperative data* :				
Cross-clamp time (min)	102 ± 43)	103 ± 42)	100 ± 45)	0.721
Circulatory arrest time (min)	21 ± 12)	20 ± 12)	23 ± 12)	0.196
CPB time (min)	205 ± 80)	220 ± 87)	190 ± 71)	0.080
Minimum temperature (°C)	25 (4)	22 (4)	28 (3)	**<0.001**
* Antegrade SCP:*	49 (53)	15 (31)	34 (77)	**<0.001**
Unilateral	10 (11)	6 (13)	4 (9)	**0.049**
Bilateral	39 (42)	9 (19)	30 (68)	**0.049**
RCP	4 (4)	3 (6)	1 (2)	0.080

Abbreviations: AV, aortic valve; CABG, coronary artery bypass grafting; CPB, cardiopulmonary bypass; MV, mitral valve; RCP, retrograde cerebral perfusion; SCP, selective cerebral perfusion; SD, standard deviation.

### Outcome and Risk Factors

During the postoperative course, renal failure ( *n* = 32, 34.8%), low cardiac output ( *n* = 26, 28.3%), and reexploration for bleeding ( *n* = 30, 32.6%) were the most common complications. Only one patient suffered from postoperative spinal cord ischemia with resultant paraparesis (1.1%). Early mortality was 44.6% ( *n* = 41) for the entire patient cohort, and worse for patients with intraoperative or catheterization-associated IAD compared with postoperative IAD (intraoperative: 43%, cardiac catheterization-related: 43%, postoperative: 27%). Fifty percent of the patients with TAVR-associated IAD died within 30 days after rescue surgery ( *n* = 3). The only significant independent risk factor for early mortality was preoperative coronary malperfusion ( *n* = 30, OR: 5.3, *p* = 0.001). Coronary malperfusion was more often present in the case of IAD due to cardiac catheterization ( *n* = 20, 67%). However, in 10 cases (33%), coronary malperfusion developed as a consequence of surgically induced IAD. Outcome data and risk factors for early mortality are listed in Table 4 .

**Table 4:** Outcomes and risk factors for mortality

	Entire cohort * n* (%)/OR (IQR)	1995–2010 *n* (%)/OR (IQR)	2010–2019 *n* (%)/OR (IQR)	*p* -Value
*Outcome* :				
Stroke	16 (17)	7 (15)	9 (20)	0.584
Sepsis	12 (13)	3 (6)	9 (20)	0.063
Renal failure	32 (35)	15 (31)	17 (39)	0.515
GI complications	16 (17)	9 (19)	7 (16)	0.788
SCI (permanent deficit)	1 (1)	0 (0)	1 (2)	0.478
Reexploration for bleeding	30 (33)	15 (31)	15 (34)	0.515
Low cardiac output	26 (28)	13 (27)	13 (30)	0.821
* Early mortality* :	41 (45)	20 (42)	21 (48)	0.675
Intraoperative IAD	23 (43)	11 (23)	12 (27)	1.000
Postoperative IAD	3 (27)	3 (6)	0 (0)	0.107
Catheter IAD	12 (43)	6 (13)	6 (14)	1.000
TAVR IAD	3 (50)	0 (0)	3 (50)	0.232
*Risk factors (early mortality)* :				
Coronary malperfusion	5.3 (2.0–13.6)			**0.001**
LVEF < 30%	4.4 (1.8–23.2)			0.079
CRF	2.5 (1.8–4.2)			0.061
Pulmonary HTN	2.1 (1.5–8.1)			0.172
NYHA III–IV	1.8 (1.2–4.2)			0.071
*Independent predictors for early mortality* :				
Coronary malperfusion	5.3 (2.0–13.6)			**0.001**

Abbreviations: CRF, chronic renal failure; GI, gastrointestinal; HTN, hypertension; IAD, iatrogenic aortic dissection; IQR, interquartile range; LVEF, left ventricular ejection fraction; NYHA, New York Heart Association; OR, odds ratio; SCI, spinal cord injury; TAVR, transcatheter aortic valve replacement.

### Changes over Time

The incidence of IAD remained comparable between early and more recent time periods (0.06 vs. 0.08%; *p* = 0.394), while patient's age significantly increased (65 ± 13 vs. 72 ± 9, *p* < 0.001). Over time the operative strategy gradually changed with antegrade selective cerebral perfusion being utilized more frequently, (31 vs. 77%, *p* < 0.001) higher minimum body core temperatures (22 ± 4 vs. 28 ± 3, *p* < 0.001) during CPB, and more frequent aortic arch repair (56 vs. 75%, *p* = 0.080; see Table 3 ). Despite these changes, outcomes remained relatively unchanged with 41.7% ( *n* = 20) versus 47.8% ( *n* = 21) hospital mortality for the early and more recent study periods, respectively ( *p* = 0.675). No statistically significant differences were found between time periods for any early adverse event ( Table 4 ).

## Discussion

Iatrogenic Type A aortic dissection is a rare but dangerous complication of cardiac procedures. Although the proportion of acute Type A aortic dissections that are IAD is estimated to be 5%, 15 the overall incidence of IAD is low, ranging from 0.14 to 0.28%. Current registry analyses 1 2 6 9 suggest an even lower rate of IAD, comparable to the incidence we found in our study. Quoted mortality of IAD—based on limited data from case series, registry data, and meta-analyses—remains high, ranging from 33 to 50%. 1 2 5 6 9

With regard to cardiac surgery-related IAD, Ram et al 2 reported that IAD occurs in approximately 0.06% of cases when the ascending aorta is the site of arterial cannulation, but 10 times more frequently when the femoral artery is used. In our cohort, the preferred site for arterial cannulation was the ascending aorta in the vast majority of patients. In cases of intraoperative IAD, we leave the arterial cannula in situ during cooling in preparation for aortic repair. 1

Although surgical outcomes for patients presenting with noniatrogenic Type A aortic dissection are slowly improving, IAD continues to be associated with high mortality. 1 16 IAD that occurs or is diagnosed during the postoperative period is associated with particularly poor outcomes. 2 While early mortality for IAD in our cohort is comparable to previous reports (45%), we found that postoperative IAD was not associated with increased early mortality rates (27%). This finding, however, may be biased due to the low number of patients presenting with postoperative IAD ( *n* = 11). Nevertheless, the presented IAD patients were in fact older than most reported non-IAD patient cohorts, which in itself is encouraging given comparable early mortality in spite of a higher risk profile.

Several authors hypothesize that a significant number of sudden deaths in patients following cardiac surgery (approximately 3–5%) are due to unrecognized IAD since autopsies are infrequently performed. 2 12 13

### Perspective on Dissection Location and Clinical Scenarios

Cardiac surgery and other cardiovascular specialties represent a heterogeneous area with a multitude of potential situations that may bear a risk for IAD. Nonetheless, our data and most of the previous reported cases are in agreement when it comes to the most frequent location of IAD. For open cardiac surgery, IAD most commonly occurs at the arterial inflow cannula (33–42%), 2 while IAD associated with cardiac catheterization most frequently originates from the right coronary artery ostium (>50%). 1 17 18 In cardiac catheterization-associated dissection, however, many patients who develop localized IAD are not referred for surgery and can be managed conservatively or by means of coronary stent implantation. 2 17 Such patients with dissections limited to the coronary sinus (Dunning's class I) or limited to less than 4 cm into the ascending aorta (Dunning's class II) 11 do not appear in most reported IAD series and are also not included in our data.

In the early years of cardiac surgery, the preferred location for arterial inflow was the femoral artery and many reports at the time described retrograde IAD as a relatively common complication (incidence of 1–2%). 2 After the introduction of ascending aortic cannulation in 1959, 19 it was observed that the incidence of IAD significantly decreased. 2 Retrograde perfusion has more recently been used with increasing frequency, as more patients undergo minimally invasive procedures. As a consequence, one may suspect that the incidence of IAD is increasing. However, neither recent case series nor our currently presented data confirm such an increase in IAD. A potential explanation may be advances in cannulation materials (e.g., smaller cannula sizes and adapted shapes) and utilization of sonography-aided implantation.

At our institution, vessel cut-down for femoral cannulation has been the preferred mode of access for minimally invasive mitral valve repair, with more recent implementation of percutaneous cannulation. Although we have performed more than 6,000 of these procedures, we have not observed an increase in IAD over time.

Although some investigators purport that IAD may be a complication unique to open cardiac surgery, 20 it is a well-recognized complication of TAVR with an incidence ranging between 0.3 and 1.9% reported in larger studies including the Placement of Aortic Transcatheter Valves trial and the German Aortic Valve Registry. 21 22 23 In our cohort—from an institution that has performed over 8,000 TAVR procedures in total—the incidence was 0.1%, which is considerably lower compared with other reports. We believe that our high center volume and subsequent experience with these procedures play a role in limiting the occurrence of IAD post-TAVR.

### Strategies to Help Minimize Iatrogenic Aortic Dissection

IAD is a complication that can require active prevention, diagnostic, and management measures from several members of the cardiovascular team. Cardiac surgeons, anesthesiologists, perfusionists, and interventional cardiologists can play a pivotal part in either circumventing this devastating complication or contributing to its timely diagnosis and treatment. 24

A plethora of measures to help minimize the risk of intraoperative IAD can be used and is the standard protocol in most cardiac centers. These measures include (1) epiaortic assessment of the ascending aorta before cannulation, (2) control of systolic blood pressure during cannulation (<100 mm Hg), and (3) verifying that the circuit line pressure is pulsatile and correlates with arterial line (radial or other) pressure before initiating CPB. To ensure safe cannulation—especially in case of utilizing the Seldinger technique for peripheral cannulation—sonography is an important tool. Both TEE and external linear transducers are helpful in ensuring correct wire placement during peripheral cannulation, and are recommended in the Guidelines for the Performance of a Comprehensive Intraoperative Epiaortic Ultrasonographic Examination from the American Society of Echocardiography, Society of Cardiovascular Anesthesiologists, and the Society of Thoracic Surgeons. 25 A detailed list of potentially helpful intraoperative measures to minimize IAD occurrence is presented in Table 5 .

**Table 5:** Intraoperative measures to minimize IAD occurrence 2

Surgeon	Anesthesiologist	Perfusionist
• Epiaortic assessment of ascending aorta before cannulation		
• Control of systolic blood pressure during cannulation and cannula removal (<100 mm Hg)		
• Caution when using aortic cannulae with stiff obturators due to risk of injury the posterior wall (e.g., in small aortic diameters)		
• Verify that the circuit line pressure is pulsatile and correlates with arterial line (e.g., radial) pressure before initiating CPB		
• Test cannula position by bolus injection and observe arterial line pressure (initial increase, prompt decrease)		
• Observing blood return/flow (>500 mL/min) via the aortic cannula to rule out occlusion or misplacement		
• In femoral artery cannulation, ensure that the guidewire is within the true lumen of the descending aorta (TEE)		
• Implement appropriate settings of an audible high-pressure alarm on the CPB arterial line (± autostop function)		
• Use of padded aortic cross-clamps or cross-clamps that generate less force		
• Lower flow or briefly halt pump flow and arterial pressure for aortic clamp application and removal		
• Cautious utilization of partial occlusion clamps. If needed, avoid applying torque		
• Consider prophylactic replacement of an abnormal ascending aorta		
• Consider using felt-reinforced over-sutures for the cardioplegia cannulation site in minimally invasive surgery (e.g., MV repair)		
• Utilization of cerebral NIRS oxygenation measurements that might indicate malperfusion due to IAD		
• Minimally invasive surgery via a right thoracotomy should be avoided in patients with an ascending aortic diameter of >4.0 cm		
• Transthoracic aortic clamping is preferable to endovascular balloon aortic occlusion for minimal invasive MV surgery		

Abbreviations: CPB, cardiopulmonary bypass; IAD, iatrogenic aortic dissection; MV, mitral valve; NIRS, near-infrared spectroscopy; TEE, transesophageal echocardiography.

### Strategies for Management of Iatrogenic Aortic Dissection

The best method of minimizing the high morbidity and mortality rate associated with IAD is of course the avoidance of this dreaded complication. However, in the rare and unfortunate event of IAD, prompt treatment is warranted and early diagnosis enables the cardiovascular team to initiate the appropriate measures.

It should be mentioned that the presented data only encompasses patients that were operated on. The number of patients with, for example, limited dissections after cardiac catheterization (e.g., Dunning I) who were treated conservatively is unknown. However, the fact that according to the records no patient with delayed IAD had a history of prior—conservatively treated—limited dissection is encouraging.

In case of postoperative IAD, we used a multimodal approach including TEE, multisite invasive blood pressure monitoring (radial and femoral pressure lines), and cerebral oxygenation monitoring via NIRS (INVOS 5100C, Somanatics Corporation, Troy, MI) to make the diagnosis. CT was used only in stable patients. In case of confirmed IAD, arterial cannulation for CPB for emergency surgery was performed via the right axillary artery.

In patients in whom IAD was diagnosed intraoperatively, the arterial cannula was left in situ (usually in the ascending aorta) and cooling was rapidly performed in preparation for aortic repair. However, this strategy requires adequate true lumen perfusion in the absence of pressure increase (arterial line) or dissection propagation. Otherwise, immediate alternative arterial cannulation should be opted for to circumvent catastrophic malperfusion and dissection exacerbation.

With regard to cerebral oxygenation monitoring, a sudden, unremitting decrease is usually addressed by immediate circulatory arrest and opening of the aortic arch to establish selective antegrade cerebral perfusion to the innominate artery and the left carotid artery (flow rate: 10 mL/kg/min). 1

The extent of aortic repair was then determined by the operative findings. In case of an isolated tear of the aortic root (frequently in the vicinity of the right coronary ostium postcatheterization), repair or replacement of the aortic root was combined with replacement of the proximal ascending aorta without arch surgery. In these and other cases with dissections involving the coronary ostia, utilization of retrograde cardioplegia may be advantageous. In many cases of IAD, the entire extent of the dissection cannot be determined immediately, which is why we advocate open aortic inspection during distal circulatory arrest. Dissections extending into or beyond the aortic arch were treated by partial or total arch replacement. Total arch replacement, however, was usually reserved for cases with the primary entry or reentry in the aortic arch upon distal inspection. Proximal aortic root repair or replacement was then performed during the rewarming phase in patients requiring aortic arch surgery (arch-first strategy). A detailed list of strategies to manage intraoperative IAD is presented in Table 6 .

**Table 6:** Strategies to manage intraoperative iatrogenic aortic dissection 1 2

Strategies to manage intraoperative iatrogenic aortic dissection
• Assess the extent of IAD by direct inspection and TEE
• Assess adequacy of: – Cerebral perfusion (TEE and/ or cerebral NIRS) – Myocardial perfusion – Severity of aortic regurgitation (TEE)
• Reduce CPB flow and arterial pressure
• In case of minimally invasive surgery or partial sternotomy—immediately change to longitudinal sternotomy for better exposure
• If detected early, consider discontinuing CPB to minimize false lumen propagation, otherwise induce cooling
• Transfer arterial inflow cannula to alternate cannulation site (e.g., femoral artery or aortic arch) and verify new cannula position using TEE—where applicable with guidewire verification in true lumen
• Move aortic cross-clamp as far distally as possible, preserving flow to the brachiocephalic trunk
• Induce deep hypothermia and consider head cooling (ice packing)
• Open the aorta and assess injury origin and extent
• Make arrangements with the perfusionist to prepare for SCP circuit
• Decide on definitive repair strategy: – Local plication – Local excision with or without a patch – Tube graft of ascending aorta – Partial or total arch replacement (± antegrade SCP) – Have a plan in case of extension into the descending aorta (e.g., frozen elephant trunk)
• Determine if it is appropriate to complete the original procedure

Abbreviations: CPB, cardiopulmonary bypass; IAD, iatrogenic aortic dissection; NIRS, near-infrared spectroscopy; SCP, selective cerebral perfusion; TEE, transesophageal echocardiography.

## Conclusion

IAD is a rare but devastating complication after conventional cardiac surgery, TAVR, and cardiac catheterization, resulting in high mortality even when an immediate emergency repair is performed. Although the patients' age increased over time and the surgical approach shifted toward more extensive arch repair, early outcomes remained unchanged in our case series.
